# New quality productivity-driven development of clinical nutrition in Chinese public hospitals: a Chongqing survey

**DOI:** 10.3389/fpubh.2026.1727081

**Published:** 2026-04-28

**Authors:** Zhongmin Gao, Xuelian Cheng, Jie Liu, Lin Kong

**Affiliations:** 1 Department of Clinical Nutrition Children’s Hospital of Chongqing Medical University, National Clinical Research Center for Children and Adolescents’ Health and Diseases, Ministry of Education Key Laboratory of Child Development and Disorders, Chongqing Key Laboratory of Pediatric Metabolism and Inflammatory Diseases, Chongqing, China; 2 Food Department of Chongqing Municipal Health Commission, Chongqing, China; 3 Department of Clinical Nutrition, The Thirteenth People’s Hospital of Chongqing, Chongqing, China

**Keywords:** Chongqing, clinical nutrition service, new quality productivity, nutritional risk screening, public hospitals

## Abstract

**Objectives:**

New Quality Productivity emphasizes innovation-driven, quality-focused development, requiring data-driven and efficient healthcare. However, Clinical nutrition is an interdisciplinary field bridging public health and clinical medicine, which is prone to marginalization and currently lacks platform development and data support. To address this, our study assesses clinical nutrition departments in Chinese public hospitals via a Chongqing survey, offering potential insights into national trends, while acknowledging regional specificity to propose data-driven solutions.

**Methods:**

The survey list of secondary and above public hospitals in Chongqing was obtained from the official website of the Chongqing Clinical Nutrition Quality Control Centre. A retrospective analysis was conducted using verified electronic questionnaire data retrieved from the National Clinical Nutrition Quality Control Platform. The collected data were organized in Microsoft Excel and analyzed by SPSS 26.0. Additionally, a targeted literature review was performed using databases such as PubMed, Web of Science, and CNKI to assess the structural, operational, and strategic challenges facing clinical nutrition departments and to formulate evidence-based development pathways.

**Results:**

The survey included 294 public hospitals in Chongqing, comprising 34.0% tertiary hospitals and 66.0% secondary hospitals. Among them, 160 (54.4%) had established Clinical Nutrition Departments, of which 42.1% had implemented the dedicated clinical nutrition information systems. These Clinical Nutrition Departments employed 643 professionals, including 284 physicians (44.2%), 187 technicians (29.1%), and 172 nurses (26.7%). This corresponds to a clinical nutritionist-to-bed ratio of 1:451. Only 11.9% of the clinical nutrition departments participated in academic education programs, whereas 33.3% engaged in clinical research activities. Between 2022 and 2024, the 24-h nutritional risk screening rate showed significant annual increases (all *p* < 0.05), and tertiary hospitals consistently achieving higher inpatient nutritional assessment rates than secondary hospitals (*p* < 0.05).

**Conclusion:**

Chongqing’s clinical nutrition sector shows progress in scale and efficacy, yet disparities between tertiary and secondary hospitals persist alongside key challenges in talent, discipline positioning, and research/education. To address these, we propose implementing the New Quality Productivity framework through national medical centers, focusing on technology integration, process optimization, and human capital development to shift from scale expansion to innovation-driven, high-quality growth.

## 1 Introduction

China’s healthcare system presented marked disparities in terms of disciplinary development (1). The therapeutic paradigm systematically prioritized biomedical interventions within the biomedical model framework, whereas multidimensional approaches incorporating nutritional interventions remained structurally marginalized (2, 3). This interregional resource allocation disparity fundamentally mirrors the era’s health policy priorities, which emphasize disease-centric therapeutic outcomes over comprehensive patient care frameworks.

**Figure 1:**
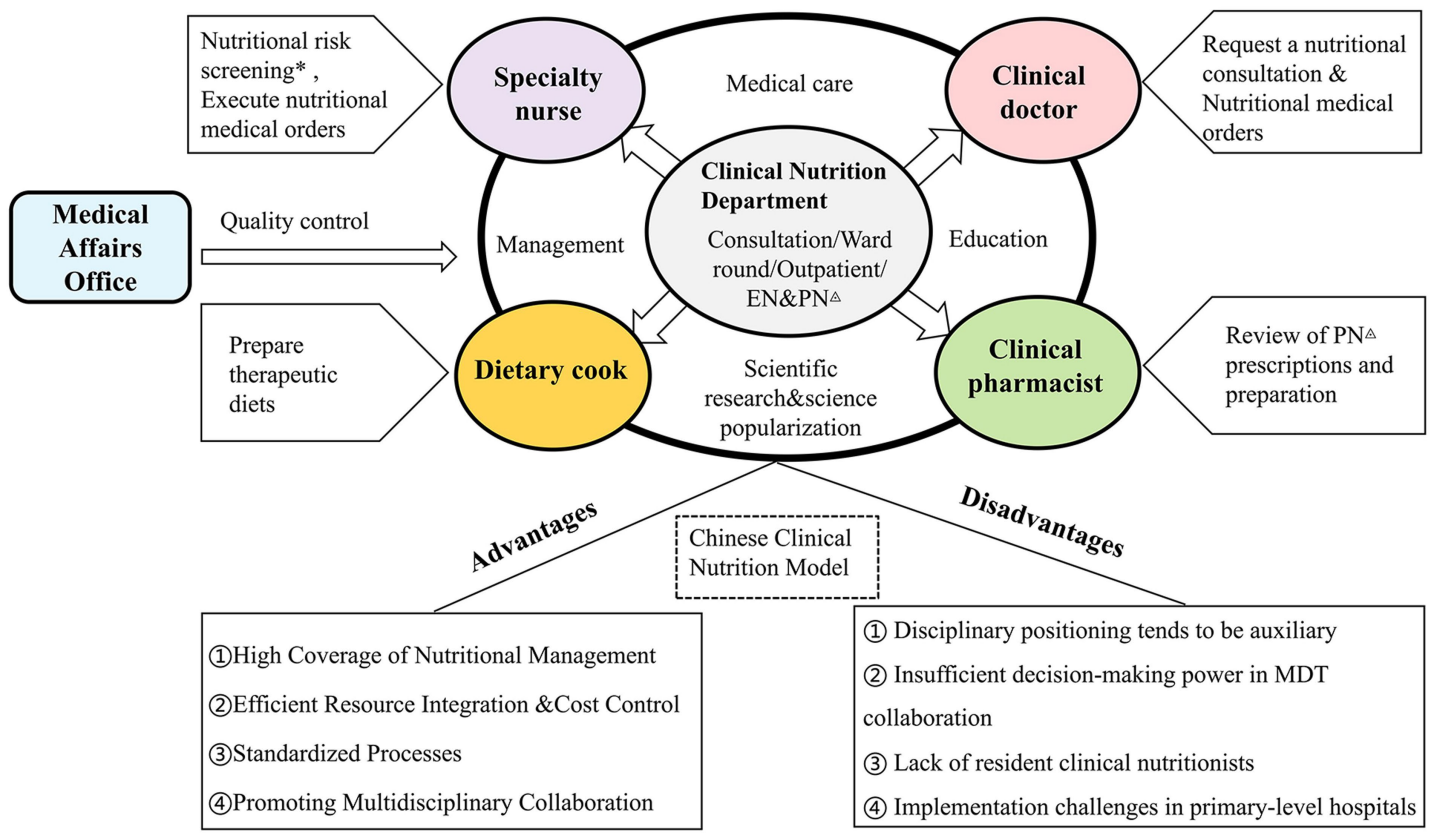
Clinical nutrition models in most Chinese public hospitals: advantages and disadvantages. * Accomplished by doctors in some hospitals; ◬PN: parenteral nutrition, EN: enteral nutrition.

However, with shifting medical models, healthcare workers now prioritize nutritional therapy. Clinical nutrition, the key to modern care, enhances patient recovery through personalized nutritional intervention (4). In China, the 2022 Guidelines for Clinical Nutrition Department Establishment and Management (trial) marked a critical milestone in the institutionalization of the discipline, mandating independent clinical nutrition departments (CNDs) in secondary and higher-level healthcare institutions with compulsory standards for personnel qualifications and service protocols (5). This top-down regulatory approach has significantly expanded CND coverage, particularly in tertiary hospitals. Nevertheless, challenges remain in primary care penetration, multidisciplinary collaboration, and service quality consistency. Although international models (e.g., nutrition support teams in Western systems) offer useful references, their operational contexts differ substantially from China’s hierarchical healthcare system. Therefore, this study focuses on the Chinese context, using the Chongqing survey to identify domestic gaps and solutions under the New Quality Productivity framework (Figure 1).

China’s New Quality Productivity (NQP) framework, introduced in September 2023, occupies a central position in the country’s current development strategy and provides a valuable conceptual framework for analyzing innovation in the healthcare sector. It shifts the focus from mere scale expansion to growth driven by quality and efficiency (6). Within healthcare, we operationalize NQP through three interdependent dimensions: (a) Technological Integration, which involves leveraging digital tools (e.g., AI, big data platforms) to enable precision medicine and intelligent management (7, 8); (b) Process Optimization, which entails redesigning clinical and administrative workflows through digital transformation and smart systems to enhance resource utilization (9); (c) Human Capital and Institutional Innovation, focusing on developing specialized talent and aligning incentives with value-based models (10). This study applies this framework to analyze the evolution of clinical nutrition services. These dimensions are reflected in the adoption of nutrition information systems, standardized care pathways, and interdisciplinary team development.

Chongqing, China’s largest municipality (32 M residents), combines urban centers, rural zones, and mountainous terrain, reflecting national urban–rural demographics and exemplifying integration duality. Chongqing’s 2023 per capita GDP (94,300 CNY) is consistent with the national average (89,358 CNY), yet sharp intraregional gaps persist: the GDP in the urban core >120,000 CNY while that of counties <50,000 CNY, mirroring China’s regional divide. Chongqing’s tertiary hospitals are clustered in nine urban districts, while primary care coverage accounts for 98%. CNDs show central strength along with peripheral neglect, mirroring nationwide public hospital resource patterns. As a national healthcare reform pilot, Chongqing explores hierarchical care and insurance models. Clinical nutrition hurdles (limited coverage, poor collaboration) mirror national gaps Chongqing’s dual traits—a “megacity scale” with only “moderate development”—make its clinical nutrition challenges a national microcosm. This study probes public hospitals’ nutritional services, identifies gaps, and proposes solutions under the New Quality Productivity program, offering a model for similar regions.

## 2 Methods

### 2.1 Survey respondents

All CNDs in secondary and above public hospitals (including comprehensive hospitals as well as specialized hospitals in oncology, pediatric care, psychiatry, and other disciplines) in Chongqing Municipality were selected as the target population for the survey.

From January 2022 to December 2024, under the Health Commission of Chongqing Municipality (HCCQ), the Clinical Nutrition Quality Control Centre of Chongqing Municipality (CNQCC-CQ) retrieved the list of medical institutions in Chongqing Municipality from the official website. A total of 294 secondary hospitals and above were identified and allocated to five district-level clinical nutrition quality control centers according to geographical distribution. Standardized training protocols were implemented across regional subcenters to enable clinical nutrition department directors to complete validated electronic surveys through the National Clinical Nutrition Quality Control Platform (NCNQCP), which served as the central data repository. The electronic questionnaire was mandatory for all identified hospitals, resulting in a 100% response rate (294/294).

This e-questionnaire was revised through literature and expert consultation, of which the contents included institutional profiles of hospitals and affiliated CNDs. Supplementary Figure shows this e-questionnaire in more detail. Prior to formal administration, a pilot test was conducted to assess the clarity and feasibility of the instrument, and modifications were made to item wording and response options based on the pilot feedback.

### 2.2 Outcome definitions

The clinical nutritionist-to-bed ratio was the ratio of the number of permanently employed clinical nutrition physicians to the number of hospital beds during the same period,it was compared against the national benchmark of 1:150, as recommended by the National Health Commission (2022) (5). The 24-h nutritional risk screening (NRS) rate was defined as the number of inpatients screened within 24 h of admission divided by the total number of inpatients admitted during the same period, expressed as a percentage (11). The inpatient nutritional assessment (INA) rate was defined as the number of inpatients who received a comprehensive nutritional assessment performed by clinical nutrition professionals during their hospital stay, divided by the total number of inpatients admitted during the same period, expressed as a percentage (11).

### 2.3 Quality control

To ensure data integrity, a dual-review verification process was established. Two investigators independently reviewed the data and, when necessary, contacted the surveyed hospitals or local quality control centers to address issues such as duplicate records, abnormal values, and missing information.

### 2.4 Statistical analysis

Data were analyzed using SPSS 26.0, with the significance level set at 5%. Normality was assessed using the Shapiro–Wilk test, which indicated a non-normal distribution (*p* < 0.05) for the variables. Accordingly, data were described as M (*p*25, *p*75). The Mann–Whitney U test was applied for comparisons among hospitals of different levels, while the Friedman test was used for comparisons across different years.

## 3 Results

### 3.1 The setup of the clinical nutrition department

A total of 294 public hospitals are located in Chongqing, with 100 tertiary hospitals and 194 secondary hospitals. As of December 2024, 160 hospitals had established CND, with a setting ratio of 54.4%, an increase of 73 compared with 2023 and 107 compared with 2022. Among them, tertiary hospitals represent 95%, and secondary hospitals represent 33.5%.

### 3.2 Personnel composition

The survey results indicate that a total of 643 professional staff members, including physicians, technicians, and nurses, are affiliated with the 160 hospitals with established CNDs. Specifically, there are 284 physicians (44.17%), 187 technicians (29.08%), and 172 nurses (26.75%). Additionally, the clinical nutritionist-to-bed ratio for CNDs in Chongqing is 1:451, which is substantially lower than the national benchmark of 1:150 recommended by the National Health Commission (2022).

### 3.3 The implementation of the key clinical nutrition work

#### 3.3.1 Nutritional risk screening rate within 24 h of patient admission

In 2022, the 24-h nutritional risk screening (NRS) rate differed significantly between secondary and tertiary public hospitals in Chongqing. Tertiary hospitals showed a higher NRS rate than secondary hospitals. In 2022, the median 24-h NRS rate was 56.22% (33.09,68.03%) in secondary hospitals and 64.29% (45.70,71.82%) in tertiary hospitals (Z = −2.064, *p* = 0.039). This disparity diminished in subsequent years and was no longer statistically significant in 2023 and 2024. From 2022 to 2024, the overall NRS rate increased consistently year by year. Both secondary and tertiary hospitals showed a similar upward trend, with statistically significant differences across the 3 years (*p* < 0.05) (Table 1).

**Table 1:** Nutrition risk screening (NRS) rates (%) within 24 h admission in secondary and above public hospitals in Chongqing.

Variables	Total (*n* = 160)	Secondary hospitals (*n* = 65)	Tertiary hospitals (*n* = 95)	*Z*	*p*-value
2024 NRS rates (%)	85.01 (64.93, 93.48)^*#^	81.02 (58.43, 92.28)^*#^	85.41 (65.27, 93.55)^*#^	−0.401	0.688
2023 NRS rates (%)	70.52 (50.19, 79.15)*	71.28 (47.64, 83.33)*	70.21 (50.22, 77.14)*	−1.553	0.120
2022 NRS rates (%)	59.61 (45.21, 69.69)	56.22 (33.09, 68.03)	64.29 (45.70, 71.82)	−2.064	0.039

*: Compared with 2022, *p* < 0.05; #: Compared with 2023, *p* < 0.05. Z-values refer to Mann–Whitney *U* tests.

#### 3.3.2 Inpatient nutritional assessment rate

From 2022 to 2024, the inpatient nutritional assessment (INA) rate in secondary and higher-level public hospitals in Chongqing showed a significant upward trend. Notably, tertiary hospitals consistently achieved higher INA rates than secondary hospitals across all 3 years (all *p* < 0.05). In 2024, the difference remained statistically significant (*Z* = −2.425, *p* = 0.015), consistent with the trends observed in 2023 (*Z* = −6.283, *p* < 0.001) and 2022 (*Z* = −5.271, *p* < 0.001) (Table 2).

**Table 2:** Nutrition assessment (NA) rates (%) among inpatients in secondary and above public hospitals in Chongqing.

Variables	Total (*n* = 160)	Secondary hospitals (*n* = 65)	Tertiary hospitals (*n* = 95)	*Z*	*p*-value
2024 NA rates (%)	7.57 (4.36, 12.44)^*^	9.49 (7.23, 11.92)^*#^	10.48 (7.62, 18.34)^*#^	−2.425	0.015
2023 NA rates (%)	7.32 (4.66, 10.67)^*^	7.61 (5.51, 9.51)^*^	11.84 (9.20, 20.32)^*^	−6.283	<0.001
2022 NA rates (%)	3.73 (2.46, 6.28)	4.22 (3.29, 5.56)	6.09 (4.77, 11.65)	−5.271	<0.001

*: Compared with 2022, *p* < 0.05; #: Compared with 2023, *p* < 0.05. *Z*-values refer to Mann–Whitney *U* tests.

### 3.4 Software and hardware

Current empirical data demonstrate that within regional healthcare institutions in 2024, 42.10% have implemented nutrition department clinical diagnosis, treatment, and management information systems, whereas 52.60% have operationalized clinical nutrition physician workstations. However, critical equipment allocation remains suboptimal: only 4 medical facilities (2.63%) possess indirect calorimetry units for precise energy expenditure measurement, and only 16 institutions (10.53%) are equipped with bioelectrical impedance analyzers for body composition analysis.

### 3.5 Teaching and academic research

Among medical institutions in the municipality in 2024, 11.92% of CNDs undertake academic instruction in clinical nutrition for tertiary education institutions, whereas 33.30% conduct scientific research.

### 3.6 Operationalization of new quality productivity dimensions

As defined in the Introduction, the NQP framework comprises three interdependent dimensions: (a) Technological Integration, (b) Process Optimization, and (c) Human Capital Development. Based on the survey data, we operationalized each dimension using measurable indicators as follows: (a) Technological Integration: adoption rate of nutrition information systems (42.1%) and availability of advanced metabolic equipment (indirect calorimetry, 2.6%; bioelectrical impedance analysis, 10.5%). (b) Process Optimization: 24-h nutritional risk screening rate [increased from 59.61% (45.21, 69.69%) in 2022 to 85.01% (64.93, 93.48%) in 2024, *p* < 0.05] and inpatient nutritional assessment rate [increased from 3.73% (2.46, 6.28%) in 2022 to 7.57% (4.36, 12.44%) in 2024, with tertiary hospitals significantly higher than secondary hospitals, all *p* < 0.05]. (c) Human Capital Development: clinical nutritionist-to-bed ratio (1:451) and the proportion of CNDs engaged in teaching (11.9%) or research (33.3%).

These indicators provide an empirical foundation for evaluating NQP-driven reforms in clinical nutrition and are directly referenced in the discussion of strategies (Section 4.2).

## 4 Discussion

### 4.1 Challenges and limitations in the construction and development of clinical nutrition departments

**Figure 2:**
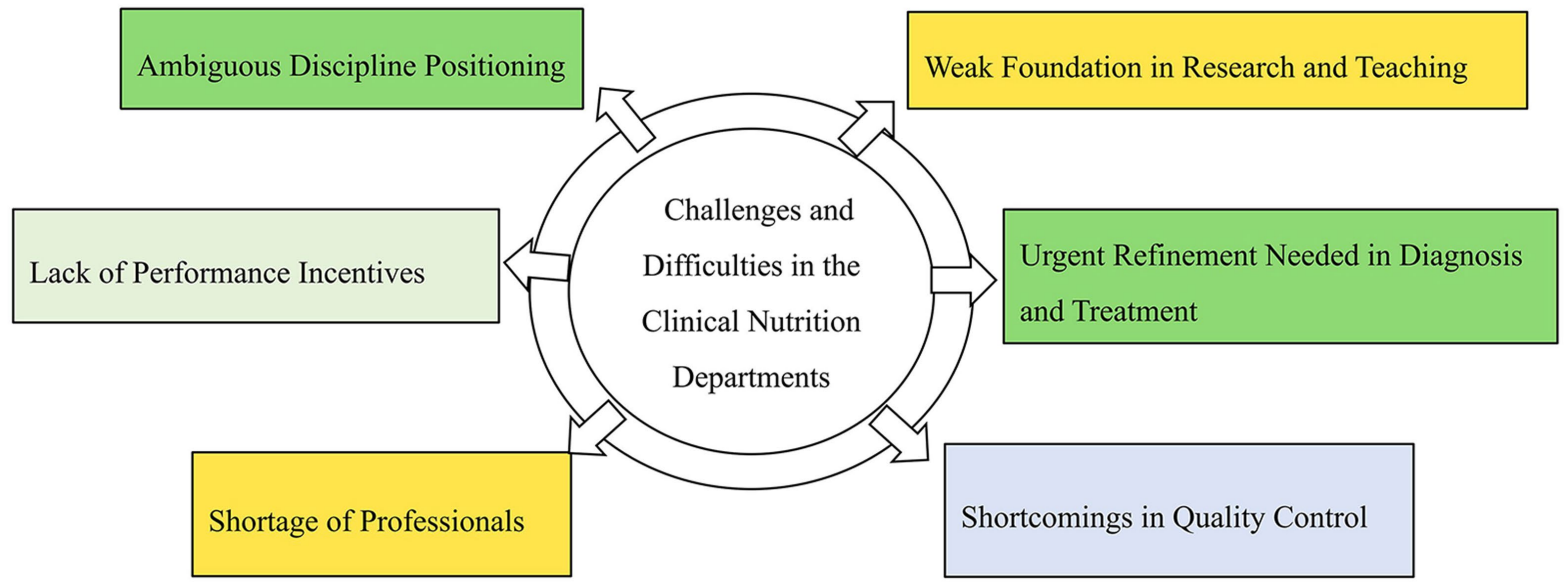
Challenges and limitations of the development of clinical nutrition departments.

The development of CNDs faces multiple challenges, including an uneven distribution of resources, nonstandardized discipline systems, and imperfect cross-disciplinary cooperation mechanisms (Figure 2).

#### 4.1.1 Challenges in disciplinary alignment and suboptimal clinical care standards

More than half of the medical institutions in Chongqing have established CNDs. Virtually all tertiary hospitals have set up such departments, and the coverage rate in secondary hospitals is also progressively increasing. This trend is consistent with the national scenario. While China’s administrative mandates drive high CND coverage, vague disciplinary roles and low prioritization persist. Current clinical nutrition services in many hospitals lack formal accreditation. A key underlying reason is that the economic potential of clinical nutrition is often underestimated (12). Studies have shown that nutritional therapy saves up to $580 million annually for patients with gastrointestinal tumors and surgical complications (13). A study by Philipson in the United States revealed that nutrition therapy saves 21.6% of medical costs (14). If China’s nutritional care matched EU/US standards (estimated to yield approximately a 20% cost reduction), projected annual savings could reach 1 trillion CNY. While top hospitals now adopt MDTs, regional disparities in implementation persist, hindering nationwide efficacy (13, 15, 16).

#### 4.1.2 Shortage of clinical nutrition professionals

A standard NST requires multidisciplinary collaboration, which necessarily involves physicians, dietitians, pharmacists, and nurses. However, the results of a previous survey revealed that many public hospitals have incomplete NST teams in China (17). This study revealed that the ratio of nutrition technicians (including nurses) to nutrition physicians in secondary hospitals and above in Chongqing is 0.8:1, whereas the ratio of CND doctors to hospital beds is 1:451, which is significantly lower than the national requirement. According to standards, the ratio of CND physicians to hospital beds should be at least 1:150, and nutrition technicians should be equipped at a 1:1 ratio with nutrition physicians (5). This finding aligns with other research reports (15, 17–19), indicating a severe shortage of nutrition professionals, particularly clinical nutritionists in secondary hospitals and above.

#### 4.1.3 Technological and digital deficits in clinical nutrition

As the clinical nutrition discipline expands its coverage, its quality has become a priority. Increasing the rate of NRS within 24 h of patient admission is the initial step toward standardizing the clinical nutrition diagnosis and treatment process (20). In 2024, Chongqing’s rate of NRS reached 85.01% (64.93, 93.48%). Nutritional assessment (NA) is the evidence-based core of clinical nutrition care, reflecting treatment quality standards (21). In 2024, Chongqing’s rate of NA for inpatients was 7.57% (4.36, 12.44%). In terms of nutritional techniques, most institutions still rely on routine enteral and parenteral nutrition therapy. Within Chongqing’s healthcare system, only four medical institutions (2.63%) are equipped with indirect calorimetry units for precise metabolic assessment, while 16 facilities (10.53%) possess bioelectrical impedance analyzers for body composition analysis. In contrast, more complex treatment techniques such as percutaneous endoscopic gastrostomy (PEG), along with detection methods like basal metabolic rate or dual-energy X-ray absorptiometry, are key measures to overcome bottlenecks in nutritional diagnosis and treatment (22).

China’s sector-wide digital progress contrasts with the lagging digitization of clinical nutrition; only 42.10% of medical institutions in Chongqing have implemented nutritional care information systems, with 52.60% operating clinical nutritionist workstations. This rate is comparable to the 47.2% adoption rate reported for secondary hospitals and above in Shanghai in 2023 (23). Moreover, some existing clinical nutrition systems operate in isolation without data integration across clinical departments, preventing nutrition specialists from conducting timely nutritional risk screening, assessment, or intervention, thereby hindering proactive nutritional therapy.

#### 4.1.4 Weak foundation in clinical nutrition research and teaching

Clinical nutrition advancement requires strong research and education crucial for professional competency. A 2016–2022 national survey revealed that declining medical institutions offered nutrition teaching, with only 33% providing student internships (24). Only 11.92% of Chongqing hospitals’ CNDs conduct teaching. Most students lack clinical internships, undermining talent development, with few tertiary hospitals engaged in nutrition education/research (17). Consistent with the findings of this study (33.30%), only one-third of tertiary hospitals reported having conducted clinical nutrition research, publishing far fewer papers than other disciplines (15, 17). Analyzing the underlying reasons, it becomes evident that only a limited number of tertiary hospitals in China are affiliated with universities and capable of undertaking teaching responsibilities at institutions of higher education. In contrast, nonteaching hospitals face systemic disadvantages in securing educational mandates. Geographically, National Medical Centers and National Regional Medical Centers are concentrated in first- and second-tier cities. These flagship institutions consistently attract high-caliber professionals through their integrated software and hardware advantages, establishing robust scientific research foundations. Conversely, primary-level hospitals exhibit distinct competitive disadvantages across these critical dimensions.

### 4.2 Effective strategies for achieving high-quality development of clinical nutrition departments in public hospitals under the concept of new quality productivity

**Figure 3:**
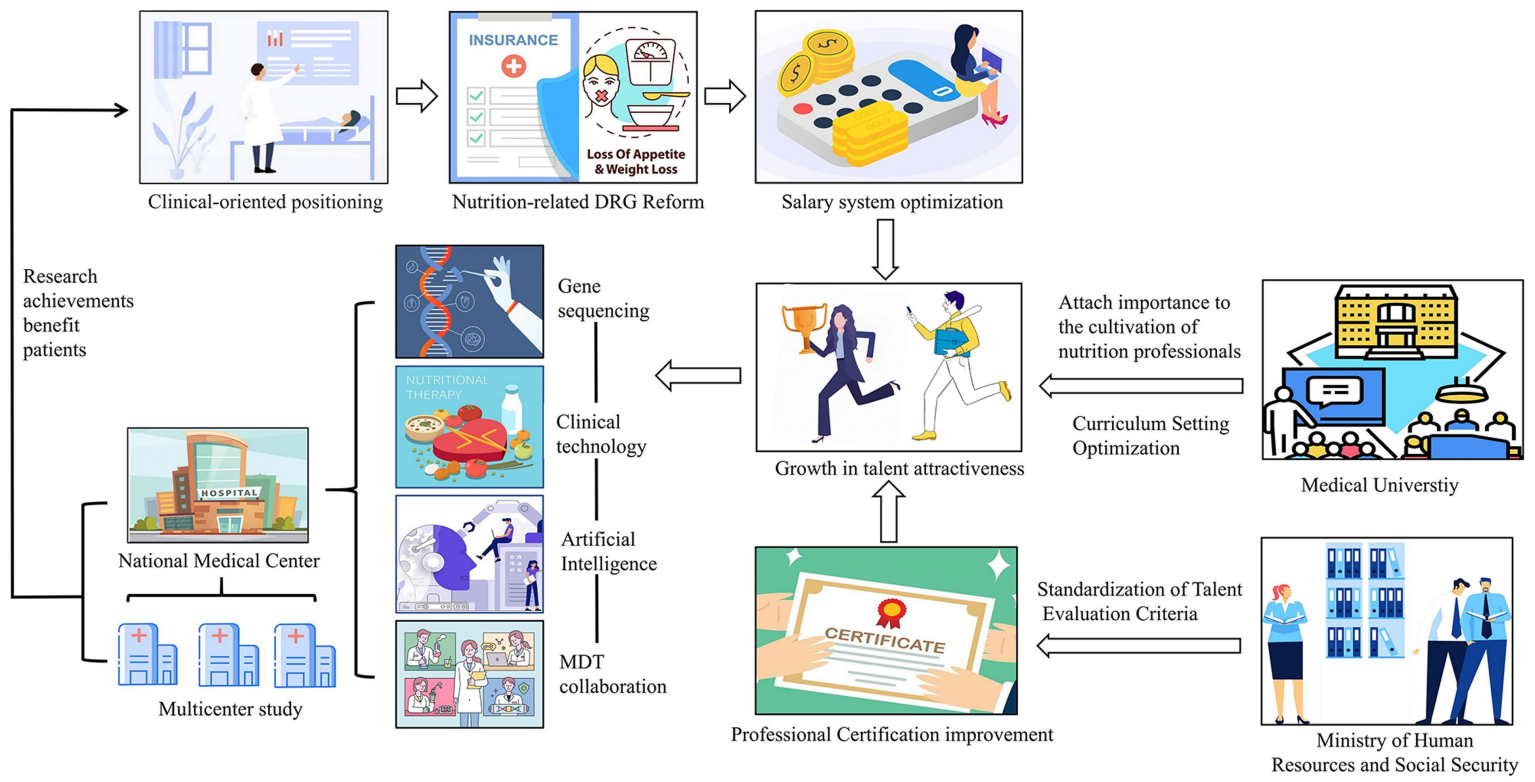
Effective strategies for the high-quality development of clinical nutrition departments in public hospitals under the concept of new quality productivity.

The high-quality development of clinical nutrition in public hospitals involves enhancing service quality, optimizing resource allocation, strengthening discipline construction, and promoting innovation to improve overall clinical nutrition. Figure 3 illustrates the logical flow of the reform and innovation methods. The three dimensions of the NQP framework were operationalized in our Results (Section 3.6) using measurable indicators from the Chongqing survey. Based on these empirical indicators, we propose the following targeted strategies.

#### 4.2.1 Bridging the digital divide: from basic integration to smart nutrition ecosystem (technological integration)

This study indicates that digital infrastructure for clinical nutrition is underdeveloped. Only 42.10% of institutions have a dedicated information system, and these systems often operate in isolation. These data highlight a primary barrier to efficient service and regional equity. We recommend that hospitals and regional health authorities immediately prioritize developing and integrating basic clinical nutrition information modules into hospital EHR systems. This foundational step enables basic data capture, improves the accuracy of quality metrics such as screening rates, and facilitates communication among multidisciplinary teams. Building on this foundation, we can pursue a visionary “smart ecosystem” leveraging AI and 5G for tele-nutrition (25).

#### 4.2.2 Advancing core nutritional techniques and precision nutrition (process optimization)

Although the 24-h nutritional risk screening rate has improved significantly [85.01% (64.93, 93.48%) in 2024], the inpatient nutritional assessment rate remains very low [7.57% (4.36, 12.44%)], with a persistent gap between tertiary and secondary hospitals. Moreover, access to essential metabolic tools, such as indirect calorimetry (2.6%) and bioelectrical impedance analyzers (10.5%), are critically limited. These results indicate that basic nutritional assessment processes are not yet standardized, and advanced phenotyping is largely unavailable. We therefore recommend that healthcare institutions first consolidate guideline-endorsed nutritional assessment protocols and ensure their consistent application, especially in secondary hospitals. Simultaneously, regional medical centers should be equipped with core metabolic measurement technologies to generate objective data for both clinical practice and research.

#### 4.2.3 Tackling workforce shortages (human capital development)

The clinical nutritionist-to-bed ratio in Chongqing (1:451) is substantially below the national benchmark of 1:150, and the support staff-to-physician ratio (0.8:1) falls short of the recommended 1:1. These quantitative deficits directly explain the low coverage of nutritional assessments and the limited research/teaching activities (only 11.9% of CNDs involved in teaching and 33.3% in research). We therefore recommend targeted measures: (a) develop dedicated recruitment plans to meet national staffing standards, starting with tertiary hospitals; (b) reform performance evaluation and compensation structures to reflect the value of nutritional care; and (c) establish transitional pathways for current practitioners to achieve standardized certification through continuing professional development.

## 5 Limitations

This study has several limitations that warrant careful consideration when interpreting the findings. First, data were collected via self-reported surveys administered within hospital departments, which may be susceptible to recall bias, social desirability bias, or inaccuracies in reporting, despite the implementation of quality control measures. Second, although Chongqing is a socioeconomically and structurally diverse municipality that reflects many of China’s urban–rural disparities, the findings may not be fully generalizable to other provinces, particularly those with differing economic characteristics or regional healthcare policies. Finally, the study did not include patient-level outcome data, which restricts the ability to assess the direct clinical effectiveness of nutritional interventions or to establish causal relationships between service delivery and health outcomes.

## 6 Conclusion

China’s clinical nutrition discipline has expanded significantly over the past 15 years, as observed in Chongqing. However, it still requires continued quality refinement. While findings from this municipal survey may reflect broader national trends, regional variations across China’s diverse healthcare landscape should be considered. To support national health goals, hospitals in similar socioeconomic contexts should address standardization gaps, talent imbalances, and weak research influence. The 2022 National Health Commission mandate calls for innovation, interdisciplinary collaboration, and international exchange to strengthen clinical nutrition’s medical, educational, and research roles, ultimately contributing to public health protection.

## Data Availability

The data analyzed in this study is subject to the following licenses/restrictions: the data supporting this study are available from the corresponding author upon reasonable request, subject to ethical approval. Requests to access these datasets should be directed to Lin Kong, bnnknn666@163.com.
