# Supplementary material for: New quality productivity-driven development of clinical nutrition in Chinese public hospitals: a Chongqing survey
Source: Front Public Health. 2026 Apr 28;14:1727081. doi: 10.3389/fpubh.2026.1727081 (PMC13161024; doi:10.3389/fpubh.2026.1727081)
Supplement: Supplementary file 1 [file Image_1.pdf]

Supplementary Figure

1.Basic information of the hospital

Q1：Hospital name： _________________

Q2：Licensed beds： _____，**Open beds：** _____

Q3：Hospital grade： ☐3A; ☐3B; ☐2A; ☐2B

Q4：Hospital type： ☐Comprehensive hospital；☐Special hospital，_________（Please fill in detail）

2.Basic information of the Clinical Nutrition Department

Q1：Is the clinical nutrition department (CND)of your hospital set up independently?

☐NO; ☐Yes

Q2：subordination:

☐Clinical department; ☐Medical and technical department ☐Logistics department; ☐Others

Q3：Personnel information of the CND：

☐Physicians_____; ☐Technicians_____; ☐Nurses_____; ☐Others_____

Q4：The area of the CND:____ m^2

Q5: Clinical nutrition physician workstations: ☐Yes; ☐NO

Q6: Nutrition Department Clinical diagnosis, treatment, and management information system: ☐Yes; ☐NO

1)The nutritional risk screening form was incorporated into the hospital information system:

☐Yes; ☐NO

2)The assessment report of nutritional status is incorporated into the hospital information system:

☐Yes; ☐NO

3. Clinical nutrition practices

Q1:Enteral nutrition preparation room: ☐Yes:_____(m^2); ☐NO

Q2:Parenteral nutrition preparation room: ☐Yes:_____(m^2); ☐NO

Q3:Medical diet preparation room: ☐Yes:_____(m^2); ☐NO

Q4:Nutritional Biochemistry Laboratory: ☐Yes:_____(m^2); ☐NO

Q5:Nutrition Clinic: ☐Yes:_____(m^2); ☐NO

Q6:Human Metabolic Phenotyping Core Laboratory:☐Yes:_____(m^2); ☐NO

Inspection, Measurement, and Test Equipment(Please fill the quantity):

☐Indirect Calorimetry System (Metabolic Cart)________;

☐Bioelectrical Impedance Body Composition Monitor________;

☐Whole-Body Physiological Function Scanner________

Q7:Clinical Nutrition Inpatient Care Unit: ☐Yes:_____(m^2),beds___; ☐NO

Q8:Clinical Nutrition Multidisciplinary Consultation Service: ☐Yes:_____(person-times); ☐NO

Q9:Charging items: ☐Yes:________(please fill the item name and price) ☐NO

Q10:Nutritional risk screening , nutritional assessment and treatment among Inpatients

1)Annual inpatient census(person-times):______

2)Nutritional risk screening(person-times):______

3)Nutritional assessment(person-times):______

4)Nutritional treatment(person-times):______

Q11:Inpatients with diabetes

1)Annual inpatient with diabetes(person-times):______

2)Nutritional assessment for diabetic inpatients(person-times):______

3)Nutritional treatment for diabetic inpatients(person-times):______

4)Diabetic inpatients who received both insulin and nutritional treatment simultaneously(person-times):______;diabetic inpatients with reduced insulin dose(person-times):______

Q12:Clinical Nutrition Research and Teaching:

1)Undertake university-level clinical nutrition teaching tasks:☐Yes; ☐NO

2)Provide clinical rotation placements for medical students from accredited higher education institutions: ☐Yes; ☐NO

3) Conduct clinical nutrition research: ☐Yes; ☐NO

4)Publication of papers: ☐Yes; ☐NO
